# Biological age of transplanted livers

**DOI:** 10.18632/aging.101378

**Published:** 2018-02-01

**Authors:** Miriam Capri, Claudio Franceschi, Matteo Cescon

**Affiliations:** 1 DIMES-Department of Experimental, Diagnostic and Specialty Medicine, University of Bologna, Bologna, Italy; 2 CIG- Interdepartmental Centre “Galvani”, University of Bologna, Bologna, Italy; 3 IRCCS, Institute of Neurological Sciences of Bologna, Bologna, Italy; 4 Department of Medical and Surgical Sciences, S. Orsola-Malpighi Hospital, Alma Mater Studiorum - University of Bologna, Bologna, Italy

**Keywords:** biological age, microRNAs, liver, transplant, donor-recipient interaction

The scarcity of human donor organs in terms of availability for transplants is a renowned problem. The high request of organs moves toward an increased use of marginal donors, including organs from old or very old donors usually transplanted into younger recipients. Within the context of orthotopic liver transplants, clinical evidence suggests that livers from aged donors (≥ 70 years) do have function and duration comparable to those achievable with livers from younger donors. Paradigmatic are the cases of 26 octogenarians livers being transplanted between 1998 and 2006, 15 patients out of 26 are currently alive and 2 of those organs being centenarians [1].

During last years, our team was deeply involved in an Italian national project (PRIN08) to collect biological data to answer the question - why livers from old donors may be successfully used for transplants. The first evidence was a relative low grade of aging signs of liver donors at histological and cytological level, also including the three major proteolytic activities of proteasome, comparing young and old livers [2]. Further, we tried to investigate the epigenetic age-related modifications in terms of liver microRNAs (miRs). We discovered that at 60-70 years of chronological age, three miRs start to increase their expression level, *i.e.* miR‐31‐5p; miR‐141‐3p; miR‐200c‐3p [3], and we assumed such an increase as markers of aging in human liver. When a relatively young liver was transplanted into a relatively older recipient (Δ age-mismatch average: + 27 years) the expression of these miRs significantly increased in the organ (follow up after graft at 15 ± 7 months). It is interesting that we were not able to document the reverse. Indeed, when a relatively old liver was transplanted into a relatively young recipient (Δ age-mismatch average: - 17 years), the expression of the three above-mentioned miRs did not change (follow up after graft at 10 ± 2 months). On the whole, these observations suggest that in the setting of liver transplantation the aging phenotype can be “transmitted/propagated” more easily than the young phenotype via the body (micro)-environment. Recently, we studied the above mentioned miRs using single-miR real time-RT qPCR on blood serum samples from 34 recipients stratified on the basis of donor liver chronological age. No difference was observed (personal unpublished data), thus suggesting that the phenomenon previously found was tightly related to the organ itself without miR-specific exocytosis and changes at circulating level, at least for the identified miRs. Thus, many other questions emerge, such as: i) the potentiality of the younger recipient on “rejuvenating” a liver from an old donor, a phenomenon that we did not observe but cannot be excluded in a larger samples and with other more informative biomarkers likely developed in the future; ii) the complexity of the interaction between the biological ages of donors and recipients, where a systemic rejuvenation effect of a young liver on the recipient should be further pursued with adequate tools. Indeed, biological and chronological age can differ substantially and chronological age is becoming an inadequate parameter to describe the health and clinical status of individuals and likely organs [4]. To this regard, we found that the biological age of the recipients, in terms of glycoage test, is older than age-matched controls [3].

**Figure 1:**
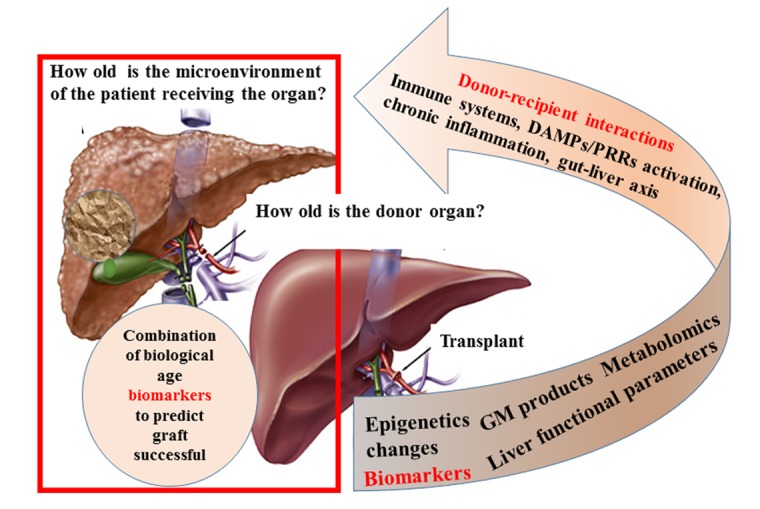
**Donor-recipient biological age mismatch**. The main scientific questions and possible answers in terms of biomarkers are focused in the context of orthotopic liver transplantation. GM = Gut microbiome; DAMPs = Danger-Associated Molecular Patterns; PRRs = Pattern Recognition Receptors.

Actually, the biological effect of donor and recipient age‐mismatch is a topic rather neglected despite its great potential, biological and clinical interest, as depicted in figure 1. The possibility that a centenarian liver can still function properly may suggest not only the intrinsic peculiarity of this organ (slowed down ageing; regeneration phenomena), but also the interaction with the younger recipients. This interaction was previously demonstrated in heterochronic parabiosis experiments in mice model at least at brain level [5], but deep analyses need specifically in humans, aiming at explain the reason of the variability associated with the duration of transplant. The need of combination of biomarkers (liver-specific functional parameters; epigenetics changes such as histone modifications, DNA methylation, tissue and circulating microRNAs; N-glycan profiles; metabolites, gut microbiome species and products) able to identify the biological ages of both donors and recipients, could be critical to explain the proper use of old or very old livers in transplant. Importantly, the combination of biomarkers should identify the biological ages at two different levels: the former at organ level (donor) and the latter at systemic level (recipient). It is expected that younger recipients may positively influence the transplant success, even if many other variables are involved besides the interactions between the different biological ages of organ and recipient, such as immune suppression efficacy, interaction between the recipient immune cells and liver donor immune cells, up to the chimera stabilization. In the liver, Kupffer cells play a central role to up-take damaged molecules originated from engraftment and enhance the response to allogenic or self-immune cells [6]. Danger molecules and DAMPs/PRRs activations are at the core of the aging process and age-related diseases [7] and similarly, the mechanisms of end-stage organ/transplant rejection maybe considered as an accelerated process of tissue/organ damage. Certainly, the individual response is the other side of the coin involving the individual-specific (personalized) immunological and cellular responses, such as repair process efficacy, remodeling and adaptation largely modulated by personal “immunobiography” [4], which may predict the final attainment of the therapy/transplant.

In the future the use of bio-engineered organs is expected, but not in a short time and not with cost accessible to everyone. In the meantime, the idea that biological age-mismatch between donor and recipient could modulate the duration of the graft at least until the complete engraftment and eventually the weaning of immunosuppressive therapy or operational tolerance seems extremely challenging. The use of donor-recipient biomarkers of biological ages and their modelling in time series studies could help the prediction of engraftment successful.
